# Continuous Aging of the Human DNA Methylome Throughout the Human Lifespan

**DOI:** 10.1371/journal.pone.0067378

**Published:** 2013-06-27

**Authors:** Åsa Johansson, Stefan Enroth, Ulf Gyllensten

**Affiliations:** 1 Rudbeck Laboratory, Department of Immunology, Genetics and Pathology, SciLifeLab Uppsala, Uppsala University, Uppsala, Sweden; 2 Uppsala Clinical Research Center, Uppsala University Hospital, Uppsala University, Uppsala, Sweden; Victor Chang Cardiac Research Institute, Australia

## Abstract

DNA methylation plays an important role in development of disease and the process of aging. In this study we examine DNA methylation at 476,366 sites throughout the genome of white blood cells from a population cohort (N = 421) ranging in age from 14 to 94 years old. Age affects DNA methylation at almost one third (29%) of the sites (Bonferroni adjusted P-value <0.05), of which 60.5% becomes hypomethylated and 39.5% hypermethylated with increasing age. DNA methylation sites that are located within CpG islands (CGIs) more often become hypermethylated compared to sites outside an island. CpG sites in promoters are more unaffected by age, whereas sites in enhancers more often becomes hypo- or hypermethylated. Hypermethylated sites are overrepresented among genes that are involved in DNA binding, transcription regulation, processes of anatomical structure and developmental process and cortex neuron differentiation (P-value down to P = 9.14*10^−67^). By contrast, hypomethylated sites are not strongly overrepresented among any biological function or process. Our results indicate that the 23% of the variation in DNA methylation is attributed chronological age, and that hypermethylation is more site-specific than hypomethylation. It appears that the change in DNA methylation partly overlap with regions that change histone modifications with age, indicating an interaction between the two major epigenetic mechanisms. Epigenetic modifications and change in gene expression over time most likely reflects the natural process of aging and variation between individuals might contribute to the development of age-related phenotypes and diseases such as type II diabetes, autoimmune and cardiovascular disease.

## Introduction

Epigenetics is used to denote the regulation of gene transcription that cannot be attributed to sequence variation in the DNA. Although the term epigenetics includes a number of different mechanisms, DNA methylation and histone modification are most commonly discussed. DNA methylation in mammals appears to be specific to cytosine, predominantly to CpG (cytosine-phosphate-guanine) dinucleotides. In promoter regions, CpG sites are often clustered in CpG islands (CGIs) were methylation is believed to repress gene expression [1]. Even small changes in the methylation of a promoter region can introduce stable changes in gene expression, leading to silencing of a gene [2], [3]. Recently, different array- and sequence-based techniques for measuring of the genome-wide DNA methylation pattern have been developed, and the different techniques have yielded concordant results [4], [5].

A number of environmental factors, such nutrition status, exposure to drugs, pesticides and other compounds, have been found to alter the epigenome [6]. A number of genomic regions have been proposed to be differentially methylated between MZ twins discordant for disorders such as schizophrenia [7], caudal duplication anomalies [8], bipolar disorder [9], and systemic lupus erythematosus [10]. More comprehensive genome-wide studies of methylation using larger sample sizes have shown that changes in DNA methylation pattern in the human brain and in blood leukocytes [11]–[13] and in cord blood from newborn compared to peripheral blood mononuclear in elderly [14], is highly correlated with the chronological age.

Although most studies performed are based on a small number of individuals or methylation sites, they point to the importance of epigenetic modifications in the process of aging. The use of novel methylation assays with a higher genomic resolution and larger sample sizes spanning a wider age-range will greatly increase our understanding of the relationship between the DNA methylation level and chronological age. In this study we evaluate the effect of chronological age on DNA methylation in 421 individuals aged 14 to 94 years, in which DNA methylation status in white blood cells has been determined at more than 475,000 sites distributed throughout the genome [15].

## Materials and Methods

### Study Population

The Northern Sweden Population Health Study (NSPHS) was initiated in 2006 to provide a health survey of the population in the Parishes of Karesuando and Soppero, County of Norrbotten, and to study the medical consequences of lifestyle and genetics. This parish has about 3000 inhabitants who meet the eligibility criteria in terms of age (>15 years) of which 1069 individuals participated in the study. Studies of populations such as NSPHS can be of great value for studying health effects of lifestyle and genetic factors. In addition, rural individuals usually live in the same environment over an extended time period, which increases the health effects of their lifestyle but also make them optimal for studying the long-term lifestyle and environmental effect on DNA methylation. For each participant in the NSPHS, blood samples were taken and immediately frozen and stored at −70 C. Genomic DNA for methylation analyses was extracted from previously frozen peripheral blood leukocytes using a phenol:chloroform protocol. More information about the NSPHS has been published previously [16]. The NSPHS study was approved by the local ethics committee at the University of Uppsala (Regionala Etik prövnings nämnden, Uppsala, Dnr 2005:325) in compliance with the Declaration of Helsinki. All participants gave their written informed consent to the study including the examination of environmental and genetic causes of disease. In case the participant was not full age, a legal guardian signed additionally. The procedure which was used to obtain informed consent and the respective informed consent form has been recently discussed in the light of present ethical guidelines [17]. Since we are working with a study cohort from a limited area, deposition of the raw data might allow for person identification. Due to concerns regarding patient privacy and protection, we are therefor unable to deposit the raw data to a central repository. However, data will be made available to researchers upon request. [17].

### Determination of DNA Methylation Status

Genomic DNA was bisulfite-converted using an EZ DNA methylation Kit (ZYMO research) according to the manufacturer's recommendations. The methylation status of the genomic DNA was then assessed using he Human Methylation450 BeadChip, (Illumina, San Diego, USA) according to the standard protocol. Analysis of the raw data was performed using Illumina GenomeStudio 2009, using the recommended settings from Illumina and the HumanMethylation450_15017482_v.1.2.bpm manifest file. The quality control (QC) parameters recommended by Illumina were used (Individual Probe Call rate >0.98, marker detection P-value < = 0.01).

### Transcription Profiling Data

Transcription profiling data from whole blood for 99 healthy US controls individuals from the PREDICT trial (http://www.clinicaltrials.gov , accession number: NCT00500617) together with information of gender and birth date were downloaded from the ArrayExpress database (www.ebi.ac.uk/arrayexpress, accessed 14 October, 2012) under accession number E-GEOD-20686. This data were produced using Agilent Whole Human Genome Microarray 4×44K 014850 G4112F, (Agilent Technologies Inc, Americas). Description on sample preparation and processing of transcription data has been published previously [18]. Gene annotations for the probes were obtained using the Ensembl database (http://www.ensembl.org, accessed –14, October 2012).

### Statistic Analyses of DNA Methylation Data

All statistic analyses were performed using the stats library of R version 2.15.0 [19]. Methylation values were reported as average beta values, which represent the ratio of array intensity signal obtained from the methylated beads over the sum of methylated and unmethylated beads (or the fraction of DNA fragments that are methylated at a specific site for an individual). Beta values for each marker were fitted to a linear model using chronological age, sex and bmi as covariables. We also tested for interaction between age and sex by using the same model and including the sex*age interaction term. No plate/batch effect were seen and consequently not included in the model. Wilcox-rank sum test were used to compare median values for not normally distributed traits. The terminology: hypomethylated is used for sites that decrease in methylation by age and hypermethylated for sites that increase in methylation by age. Bonferroni adjustment for multiple testing was used when determining if a site was hypo- or hypermethylated. Modal clustering was performed for each site separately using the Modalclust library [20], without any assumptions of the underlying number of modes and memberships and using a smoothing factor of 0.05. To compare the number of modes with the presence of genetic polymorphisms, SNP information were downloaded from the 1000 Genomes reference panels representing the 23 Nov 2010 (low-coverage genomes) and 21 May 2011 (high-coverage exomes) accessed from the IMPUTE-web resource (http://mathgen.stats.ox.ac.uk/impute/data_download_1000G_phase1_integrated.html) March21, 2013. Only 1000 Genomes SNPs with a minor allele frequency of 0.01 in European populations were included in the analyses.

### Statistic Analyses of Transcription Data

Linear regression was performed for transcription data using chronological age and sex as covariables. For the transcription data, a cut-off of P = 0.05 were used to determine if the gene expression increased/decreased by age. Chi-square test were used to test for enrichment of genes that increase or decrease in expression by age among the most hyper- and hypomethylated genes relative other genes.

### Functional Analyses of DNA Methylation Sites

Annotation of DNA methylation sites were provided by Illumina (www.illumina.com, HumanMethylation450_15017482_v.1.1.csv, accessed: 1^st^ September, 2012) as described previously [15] or downloaded from the UCSC genome bioinformatics site (http://genome.ucsc.edu, Feb. 2009 (GRCh37/hg19) release, accessed: 1^st^ September, 2012). The definition of a CGI used were: length >200 bp, GC content > = 50% or greater observed to expected number of CG dinucleotide >0.6. CpG sites located in Island shores are located 0–2 kb and shelves 2–4 kb from an island. N shore and shelves are located upstream and S shores and shelves are located downstream of an island. Chromatin states in blood cells [21] were downloaded from the UCSC genome bioinformatics site (http://genome.ucsc.edu, Feb. 2009 (GRCh37/hg19) release, accessed: 1^st^ September, 2012).

### Selection of the most Hyper- and Hypomethylated CGIs

Each autosomal CGI with more than three measured CpG sites (N = 25045) were analyzed for fraction of sites were the methylation level was positively (correlation coefficient >0 and Bonferroni adjusted P-value <0.05) or negatively (correlation coefficient <0 and Bonferroni adjusted P-value <0.05) correlated with age. The CGIs were ranked according to the fraction of sites in each category (hypo- or hypermethylated). The top 500 were included in the gene ontology analyses. Gene annotations for the CGIs were provided by Illumina (www.illumina.com, HumanMethylation450_15017482_v.1.1.csv, accessed: 1^st^ September, 2012).

### Biological Function and Gene Ontology Analyses

Gene ontology analyses were performed using GORILLA (Gene Ontology enRIchment anaLysis and visuaLizAtion tool) [22] for autosomal markers only. A CGI might not be linked to any known gene or be linked to a number of different genes with similar functions. To avoid introducing a bias from genes of similar function tending to cluster to the same chromosomal location, only one gene for each CGI was randomly included in the final gene lists of 500 candidates.

## Results

**Figure 1:**
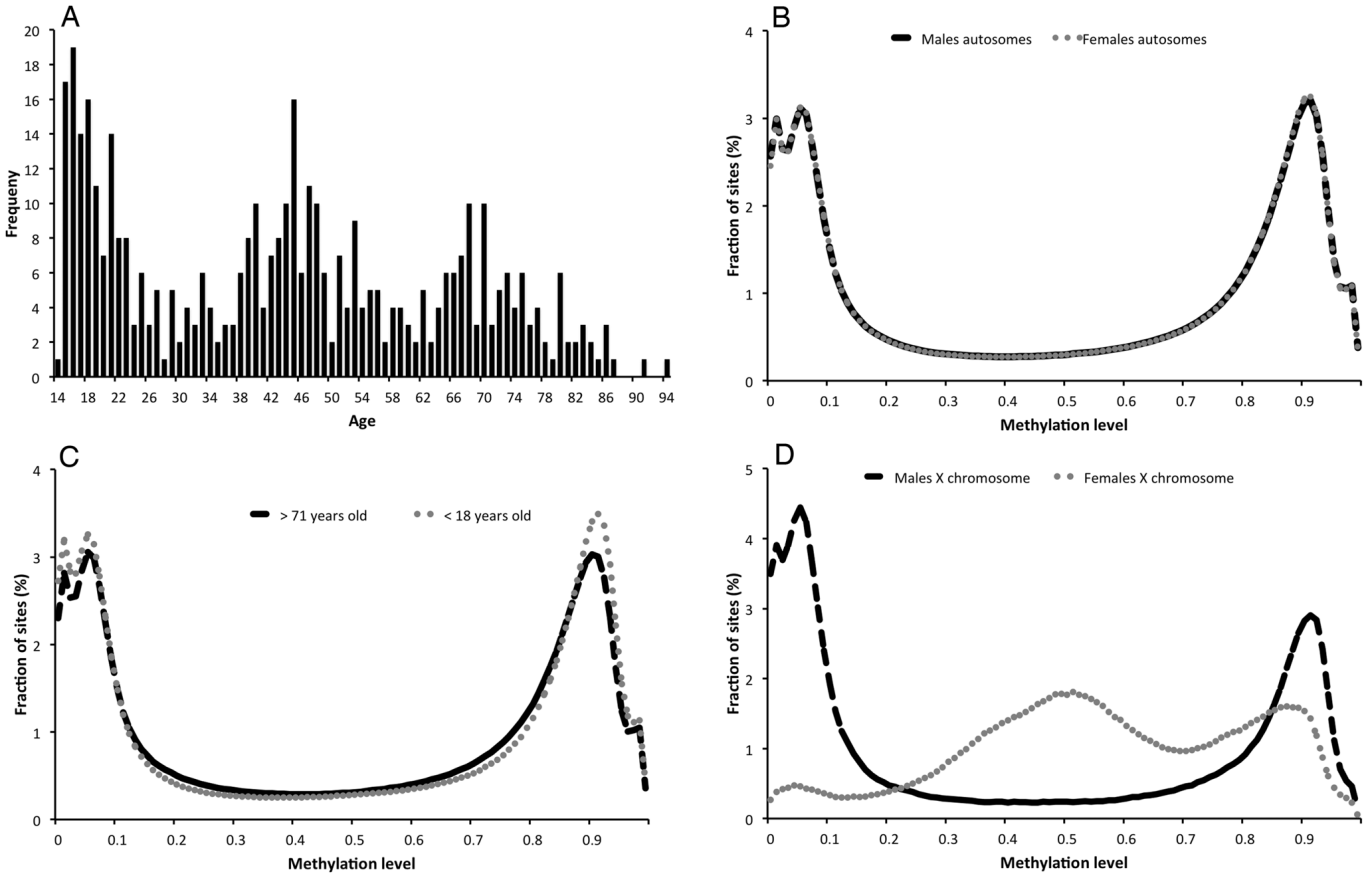
Distribution of A) Ages in the study cohort, B) DNA methylation levels for autosomal markers in males and females, C) DNA methylation level for autosomal markers in the youngest (age <18, N = 51) and oldest (age>71, N = 52) individuals of the study, and D) DNA methylation levels for X chromosomal markers in males and females.

A total of 476,366 markers (98.10%) and all individuals (N = 421, Probe Call rate ranging from 99.72% to 99.99%) passed the QC. The median age among individuals was 44 years, ranging from 14 to 94 (Figure 1A). The distribution of DNA methylation levels shows, that among autosomal markers, as many sites are unmethylated (methylation level <0.25) as are methylated (methylation level >0.75) (Figure 1B and C). The distribution of DNA methylation levels differs somewhat between males and females for the autosomes (Figure 1B), with a slightly lower median autosomal methylation level for males (p-value = 0.031). No difference in quantile or mean autosomal methylation levels is seen between sexes. By contrast, the pattern for the X-chromosome differs dramatically between sexes (Figure 1D). This is in agreement with imprinting of a large fraction of the X chromosome in females giving rise to a large fraction of hemi-methylated sites in females (methylation level >0.3 and <0.7) in combination with the pseudo autosomal region of the X-chromosome with unmethylated (methylation<0.25) or methylated (methylation >0.75) sites also in females. The methylation pattern of autosomes differs between the youngest and oldest participants in our study (Figure 1C). The mean autosomal methylation level decreases with age (R = −0.191, p-value = 8.05e-05), but the most significant difference is the increase of the 1^st^ quantile (R = 0.406, p-value <2.2E-16) and decrease in median (R = −0.439, p-value <2.2E-16) and the 3^rd^ quantile (R = −0.429, p-value <2.2E-16), similar to that seen in Figure 1A.

### Modal Clustering

A number of factors might influence if the distribution of methylation levels will be unimodal, bimodal or polymodal (including multiple distributions with different mean/median). The most obviously pattern is the imprinted part of the X chromosome, were most males will appear to be unmethylated compared to female who are hemimethylated at most sites (Figure 1D). In this case we expect a bimodal distributions for most X chromosomal sites, with the mean of the first mode (males) being close to 0 (unmethylated) and the second mode (females) being close to 0.5 (hemimethylated). Another scenario is the case of having an SNP in the CpG site. A polymorphism in one of these positions might lead to poor or no methylation. Also SNPs located within the sequence of the DNA probe used for genotyping might affect the specificity of a probe and differences in intensities between different alleles. Most of the autosomal sites (94.7%) in our data are monomodal (Table 1). However, a number of sites are bimodal (4.6%), whereas only few sites were trimodal (0.74%), or had more than three modes (0.023%). Unfortunately, we do not have complete SNP information for the regions surrounding the DNA methylation sits. However, we can determine that a significantly (P-value = 3.3e-246) higher fraction (28.1%) of the trimodal sites overlap with a known 1000 Genomes SNP located at the DNA methylation site, compared to only 6.5% for the bimodal sites and 0.5% of the unimodal. Similarly, a significantly (P-value = 7.8e-148) higher fraction of bimodal trimodal sites (45.4%) overlap with a 1000 Genomes SNP located within 1 bp from the DNA methylation site measured, as compared to 7.5% for the bimodal and 0.75% for the unimodal. However the frequency of sites with an SNP located 2 bp from the DNA methylation site measured is more similar between trimodal, bimodal and unimodal sites (0.75%, 0.72% and 0.45% respectively). This suggests that underlying SNPs might account for a large fraction of the variation in the bi- and especially tri- modal DNA methylation sites.

**Table 1:** Number of modes in the distribution of DNA methylation for each site.

Number of modes	1	2	3	>3
Number of Sites (N)	451002	21707	3546	111
Markers with a known SNP within 10 bp from the site	0.067	0.113	0.325	0.360
Markers with a known SNP more than 10 bp away from the site	0.123	0.115	0.120	0.189
Markers with a known SNP within or more than 10 bp away	0.178	0.205	0.368	0.432
Markers without any known SNP within or more than 10 bp away	0.822	0.795	0.632	0.568

### Principal Component Analyses

Principal component analyses of all autosomal markers show that only the first four principal components (PCs) each explains more than 1% of the variance among the beta values (61,1%, 20.3%, 4.2%, and 1.4% for PC 1–4 respectively). All other PCs explain less than 1% of the variation each. Together the first four PCs account for 87.1% of the total variance in DNA methylation (Table 2). All the first four PCs are strongly correlated (p<2.65E-06) with chronological age, with a Rhô2 ranging from 0.051 to 0.278. To estimate the fraction of variation in DNA methylation that can be attributed age, we extrapolated these numbers. Since age explains Rhô2 = −0.528?2 = 27.9% of the variation in PC #1 and PC #1 explains 61,1% of the variation in the DNA methylation, we can estimate that age explains 27.9% *61.1% = 17% of the variation in DNA methylation based on only looking at PC #1. If calculating the sum of these variances for PC1–4, we can estimate that age account for more than 23% of the variation in methylation level among the autosomal sites.

**Table 2:** Principal components for the DNA methylation levels among autosomal markers and corresponding correlation with age and variance.

Principal component	Proportion of variance explained by each PC	Cumulative proportion of the variance	Spearman's rank correlation with age		Total variance attributed by age*	Cumulative variance attributed to age
			Rho	P-value		
PC1	0.611	0.611	−0.528	1.84E-31	0.170	0.170
PC2	0.203	0.814	0.534	2.68E-32	0.058	0.228
PC3	0.042	0.856	−0.387	1.94E-16	0.006	0.234
PC4	0.014	0.871	−0.227	2.65E-06	0.001	0.235
PC5	0.009	0.879	0.305	1.84E-10	0.001	0.236
PC6	0.004	0.884	0.153	1.64E-03	0.000	0.236
PC7	0.004	0.887	−0.049	3.16E-01	0.000	0.236
PC8	0.003	0.890	0.108	2.67E-02	0.000	0.236
PC9	0.002	0.892	−0.287	2.13E-09	0.000	0.236
PC10	0.002	0.894	−0.163	7.82E-04	0.000	0.236

* Total variance attributed by age is calculated as Rhô2 * the proportion of variance explained by the PCs.

### DNA Methylation and Correlation with Chronological Age

**Figure 2:**
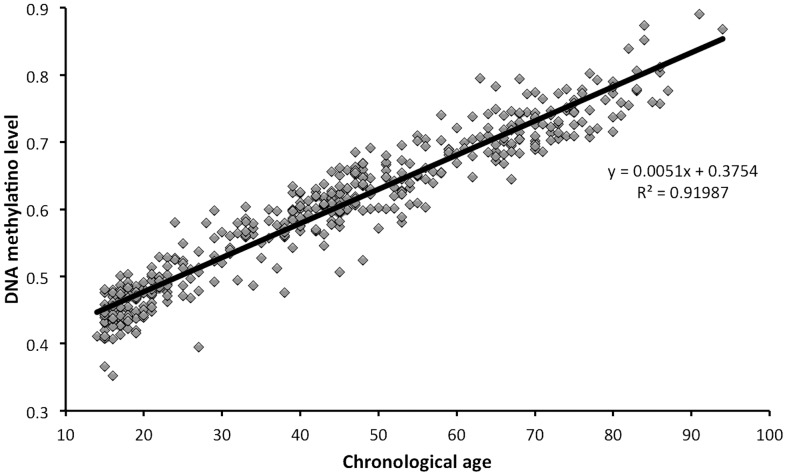
Increase in DNA methylation level with age of one CpG site (cg16867657) in the promoter of the ELOVL2 gene and corresponding regression line.

For as many as 29.0% of the sites (137993), the methylation level is significantly (P-value <1.049613e-07, Bonferroni adjusted P-value <0.05) correlated with age (Supporting information Table S1). For the majority of these sites (60.5%) methylation decrease (hypomethylation) with age and for remaining (39.5%) methylation increase (hypermethylation). For 37.8% of all sites, the methylation level was not correlated (Nominal P-value >0.05) with age. For most sites influenced by age, DNA methylation increase/decrease approximately linear with age. The strongest positive correlation of methylation with age is seen for cg16867657 (R = 0.957, P-value = 1.20e-228), which is located in a CGI in the promoter of ELOVL2 (Figure 2), were the methylation level ranges from 0.352 in the youngest to 0.891 in the oldest individual. It is interesting to note that the distribution of methylation levels for the cg16867657 site were estimated to be bimodal with means u = 0. 491 and u = 0.625, as a consequence of the uneven distribution in age among individuals and an increased number of individuals with an age around 17, 45 or 67 years of age (Figure 1A). In addition to being age-dependent, significant interactions between sex and age was observed (Bonferroni adjusted P-value <0.05) for 163 sites, of which 152 were located on the X chromosome. This low number of significant interactions indicates that the effect of age on DNA methylation is similar in males and females for most sites.

### Functional Analyses of Marker Locations

**Figure 3:**
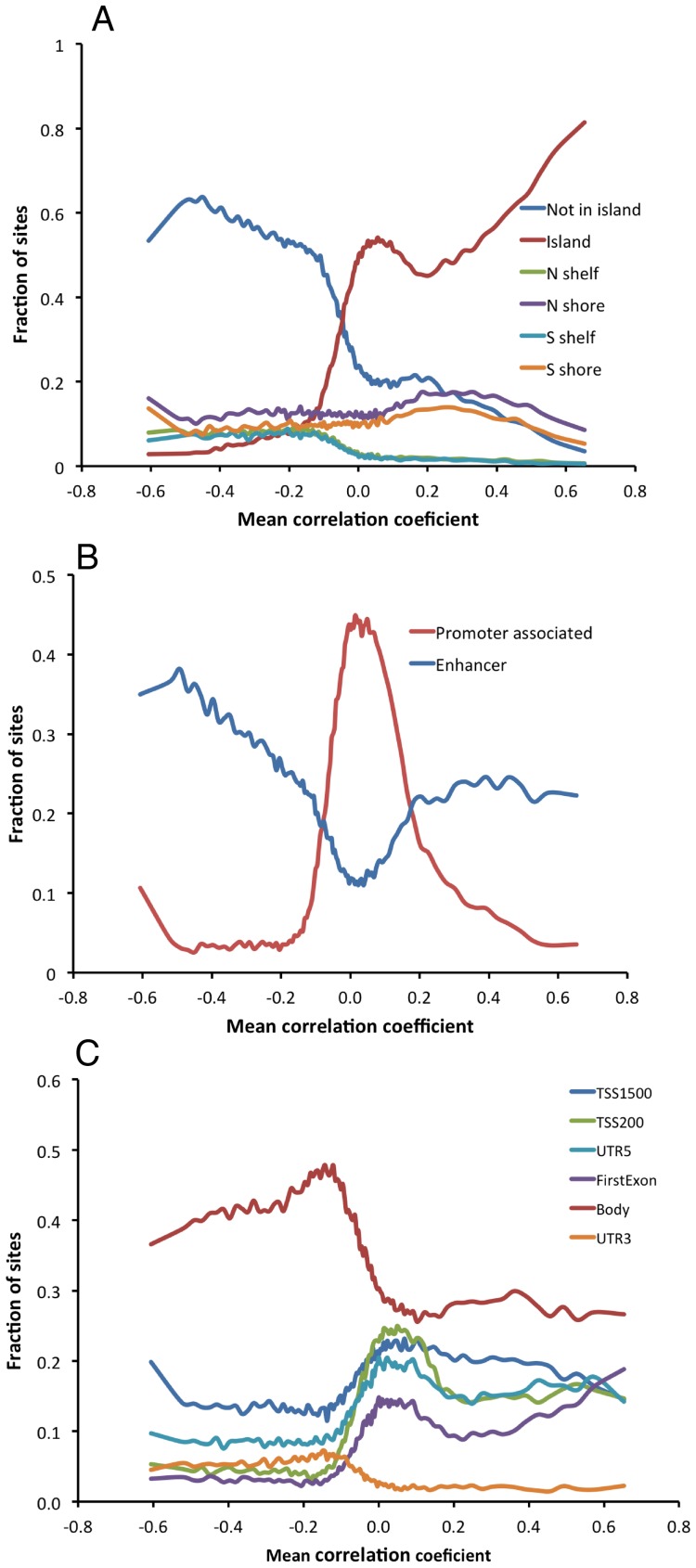
Location of CpG site depending on correlation between DNA methylation level and chronological age. Observations are ordered by the correlation coefficients and combined into 100 bins. The illustrations show the fraction of markers within each bin with a location in relation to, A) CGIs, island shores and islands shelves, B) Known promoter and enhancer regions, and C) Gene and transcription starting site.

**Figure 4:**
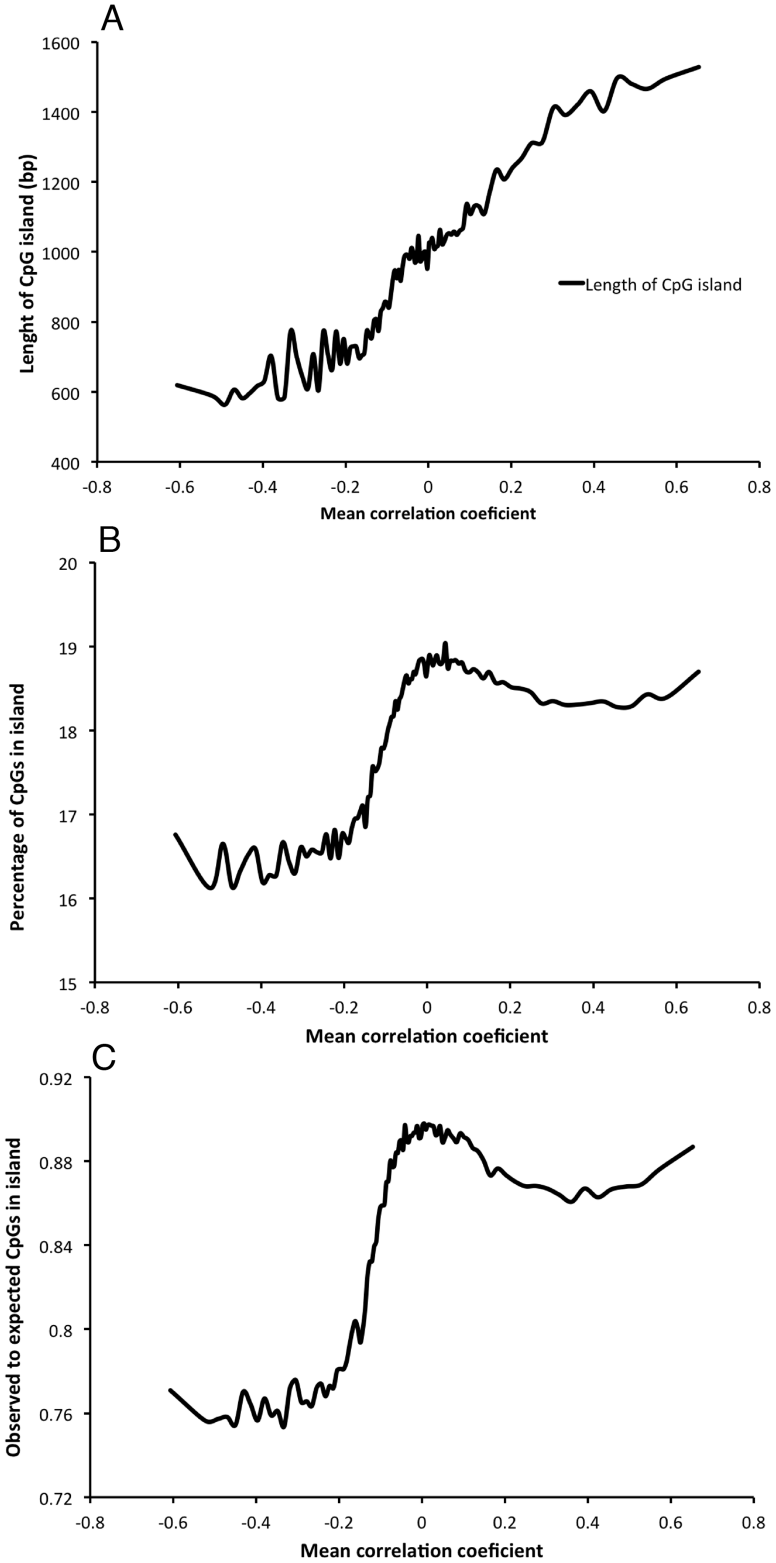
Summary statistics for the CGIs depending on the correlation between DNA methylation level and chronological age. Observations are ordered by the correlation coefficients and combined into 100 bins. The features of the CGIs within each bin is summarized as, A) Mean length of the CGIs, B) Mean percentage of CpGs in the islands, and C) Mean of observed to expected ration of CpGs in the islands.

The Human Methylation450 BeadChip is designed to target more or less all well-defined CGIs in the human genome. In total, 27110 CGIs are targeted with at least one of the markers that passed the QC in our analyses. In total, 304217 (63.9%) of the markers overlap with a CGI, with an average of 17.5 markers (median = 10) per island, ranging from 1 to 166 markers. As many as 21153 genes are targeted by at least one CpG site, 15930 by a CGI and 10128 genes have at least one CpG site in the promoter. Among the sites that become hypermethylated with age, 81.4% are located in a CGI, compared to only 2.8% for the hypomethylated sites (Figure 3A). On the other hand, CpGs located in S shelf or N shelf, very rarely become hypermethylated by age, whereas the frequency of S shore and N shore sites is more evenly distributed over the spectra of correlation coefficients. This agrees with previous studies using another chip (Infinium HumanMethylation27 BeadChip) with larger fractions of CGIs, showing that a larger fraction of sites become hypermethylated by age [13]. While the fraction of sites located within CGIs increase with increasing correlation coefficient, there is also a peak representing a large fraction of sites in CGIs with a correlation coefficient close to zero (Figure 3A). This peak is mainly due to promoter sites (Figure 3B), which appear to be more conserved in the DNA methylation level throughout the years compared to e.g. enhancer sites. The same peak is also found for sites located 200 bp upstream of the transcription starting site, 5′ UTR and the first exon, whereas a larger fraction of the sites are located in gene bodies and 3′UTRs appear to become hypomethylated over time (Figure 3C). It is also seen that longer CGIs more often become hypermethylated by age (Figure 4A), and that the fraction of observed CpG sites compared to the content of C and G nucleotides is highest for sites that are not correlated with age and sites where the methylation level is positively correlated with age (Figure 4 B and C).

**Figure 5:**
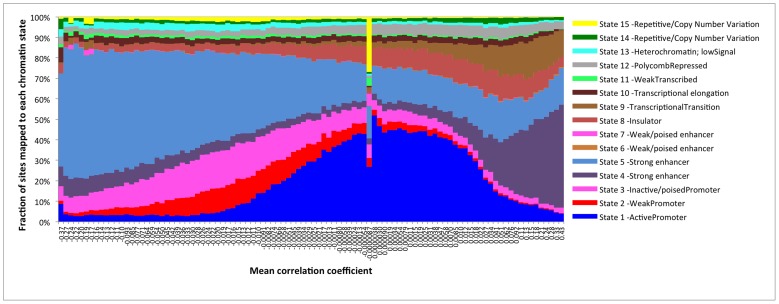
Distribution of DNA methylation sites mapped to regions with different chromatin states as defined by Ernst et al [**21**] **.** DNA methylation marks are ordered by the correlation coefficient with age and combined into 100 bins.

Ernst and Kheradpour [21] define, in blood cells, two types of strong enhancers; chromatin state 4, which is defined by locations having H3K4me1/2/3 and acetylated H3K27 and H3K9, and chromatin state 5, which is defined as having H3K4me1/2, acetylated H3K27 and to some degree acetylated H3K9. While DNA methylation in chromatin that has been annotated as being active promoters (state 1), appears to be less affected by age in our data, we find that strong enhancers of state 4 is predominately found among hypermethylated sites and enhancers of state 5 among hypomethylated sites (Figure 5).

### Gene Ontology Analyses

The genes with hypermethylated CGIs are overrepresented in some molecular functions and cellular processes. The most enriched molecular functions involve DNA binding and transcription regulation, which are enriched almost eight times (P-value down to P = 9.14E-67) for hypermethylated genes (Supporting information Figure S1 and Table S2). Similarly, a large number of biological processes are enriched for hypermethylated genes (P-values down to 3.19E-49), including processes of anatomical structure and developmental process, pattern specification process, and regionalization and neurological functions. For example, cerebral cortex neuron differentiation is over 30 times enriched for hypermethylated genes (Supporting information Table S3, and Figure S2). The genes that become hypomethylated with age do not show any strong enrichment for specific functions or processes (Supporting information Table S4, Table S5, Figure S3 and Figure S4).

### Gene Expression and Chronological Age

We wanted to investigate if genes that become hyper- of hypomethylated also show a more distinct change expression with chronological age. A total of 28984 out of 41049 expression probes mapped to a gene with an official gene name provided by Ensembl and could be mapped to our most hypo- or hyper-methylated gene associated CGIs. Among the 500 most hypermethylated CGIs, 412 are associated with a gene that is also targeted by at least one expression probe. Similarly, among the 500 most hypomethylated CGI, 407 are associated with a gene that is also targeted by at least one of the expression probes. Among the 28984 probes that are mapped to a gene name, a slightly higher fraction (16.0%) decrease compared to increase (13.3%) expression with age (p = 2.29e-21). For the genes that become hypermethylated by age, a much larger fraction (26.94%) decrease in expression with age and a much smaller fraction increase in expression by age (2.91%) compared to the average (Chi2 test P-value = 2.49e-09 and 6.55e-10 respectively). Interestingly, also for genes that become hypomethylated by age, a much larger fraction (24.8%) decrease in expression with age and a much smaller fraction increase in expression by age (6.88%) compared to the average (Chi2 test P-value = 1.807e-06 and 0.000158 respectively). These results suggest that both hyper- and hypo methylation by age can result in a decrease of expression, probably by different regulatory mechanisms.

## Discussion

In this study we have shown that chronological age plays an important role in the pattern of DNA methylation in white blood cells. More than 23% of the total variation in autosomal DNA methylation, or over 90% of the variation at individual sites, can be attributed to chronological age. As many as 29% of the sites surveyed were influenced by age using a stringent cut-off for significance (Bonferroni adjusted P-value <0.05). Our estimate is higher compared to previously studies [11], [12], mainly due to the larger number of sites investigated, the larger sample size, and the wide range in age between subjects. The distribution of mean methylation level per site also differs between age-stratified groups. It appears that in younger individuals a larger fraction of sites are either hypo- or hypermethylated, whereas for older individuals sites that were initially hypomethylated increase in methylation level, and sites that were initially hypermethylated decrease in methylation level. This indicates that there is a change in methylation level with age, from an initial hyper- or hypomethylated state and towards hemimethylation. Interestingly, a lower number of sites appear to become hyper- as compared to hypomethylated by age.

For a large fraction of the sites (38%), the methylation level did not correlate with age (nominal p>0.05). Many factors might result in lack of a correlation, including methodological issues (typing quality), strong influences on the methylation level by other (as yet unidentified) factors (e.g. diet and lifestyle), and bi- or polymodal distributions (e.g. due to SNPs). In addition, we do observe a higher fraction of sites in repetitive regions (chromatin state 15) that are not affected by age (Figure 5) suggesting that many of theses sites might not be related to gene regulation. However, it is reasonable to believe that some sites might be subjected to strict conservation of the methylation pattern throughout the lifetime. Interestingly, we observe a peak of sites with correlation coefficient close to zero to be enriched for sites located in CGIs (Figure 3A) and in active promoters (Figure 3B and Figure 5). This might suggest that the DNA methylation pattern of promoters is more conserved through life and that changes in the methylation pattern of promoters are more likely to be associated with a phenotype in humans.

There is a clear overrepresentation of specific biological functions among hypermethylated genes compared to almost no overrepresented functions of processes among hypomethylated genes. Together these results indicate that hypermethylation with age is more site- and gene-specific, and that hypomethylation is likely to occur more sporadically at sites with a less central role in regulating gene transcription. This agrees well with previous studies showing that the methylation level correlates with chromatin accessibility, and that the most variably methylated CpGs are often found in gene bodies and intragenic regions rather than in promoters and upstream regulatory regions. While DNA methylation of promoters is generally regarded as repressing gene expression, methylation of genic regions has been correlated to increased transcriptional activity [1]. This might explain our observation that the pattern of more stable methylation or hypermethylation by age in regions close to the transcription start site, as compared to the hypomethylation seen at sites in gene bodies (Figure 3). This pattern suggests that transcription at many genes is reduced with increased chronological age, which agrees with our observation from transcription profiling data, where both hyper- and hypermethylated genes are enriched for lower transcription levels by age.

The change in methylation pattern is dependent on the balance between modifying and demodifying enzymes. Most of the enzyme activity in the process of copying the methylation pattern during replication is attributed to the DNMT1 methyltransferase. Other known components in DNA methylation pattern are *de novo* methyltransferases, DNMT3A and DNMT3B [23]. The mechanisms of DNA methylation also agree with hypomethylation being a failure (which might be more pronounced at some sites) to copy the methylation pattern, while *de novo* methylation acts in a site-specific manner. The fact that the average gene expression level decrease by age and that decreased levels of DNMT1 and other enzymes has been shown to result in genome-wide loss of CpG methylation [24], support our results of the larger number and less site specificity of hypomethylation.

Another functional difference between sites that become hypo- or hypermethylated by age is that hypermethylated sites more often map to regions that have been suggested to represent strong enhancer of chromatin state 4, as compared to hypomethylated sites that mainly map to strong enhancers of chromatin state 5 in blood cells (Figure 5). State 4 and state 5 strong enhancers mainly differ by state 4 having trimethylated lysine 4 of histone H3 (H3K4me3) and acetylated lysine 9 of histone H3 at (H3K9ac) [21]. Our results of a positive correlation between hypermethylation by age and H3K4me3 in blood cells agree with other studies [12]. However it has previously been shown that H3K4me3 inhibits the binding and activity of the *de novo* methylation enzymes DNMT3A/B [25]. This does not agree with our results of state 4 enhancers (being H3K4me3) being more prone to hypermethylation. However, it has also been shown that both H3K4me3 and H3K9ac decrease with age, at least in brain tissue [26], [27], which could result in higher activation of DNMT3A/B followed by hypermethylation of state 4 enhancers, which does agree with our results. It is of interest to note that the gene ontologies that were enriched for H3K4me3 methylation specific to newborns [27] overlap with the ontologies that we find to become hypermethylated by age. Also, out of 557 genes that were H3K4me3 specific to the newborn, a significantly larger fraction (5.9% compared to 2.7%) overlap with the genes becoming hyper- as compared to hypomethylated by age in our study (Fisher Exact Test one tailed P-value = 0.0045). Similarly, out of 92 genes that are H3K4me3 specific at higher age [27] five overlapped with our hypo- and none with our hypermethylated genes (Fisher Exact Test one tailed P-value = 0.030). The age-related changes in histone modification and the association between age-dependent histone modifications and age-dependent DNA methylation are poorly understood [28]. Our results indicate that the epigenetic mechanisms that reduce gene expression throughout our lifespan are shared between different tissues. In addition, our results suggest that the changes in the epigenetic pattern by chronological age is to some extent correlated between DNA methylation and histone modifications. One explanation could be that for some genes that become less active with age, H3K4me3 decrease followed by enhanced *de novo* DNA methylation.

Compared to most studies on DNA methylation, which are performed in vitro on specific cell types, our study was performed on white blood cells (WBC) from peripheral blood samples from a population-based cohort. WBC represent a mixture of cell types and we cannot exclude that some of our findings are due to age-dependent change in the composition of different cell-types rather than average change in DNA methylation over all cell-types. It has been shown that DNA methylation vary depending on cell composition, an effect that is pronounced in immune system-related diseases [29], [30]. In previous studies, the white blood cell counts (WBC) for the most common cell types (neutrophils, eosinophils, monocytes, and lymphocytes) have been compared to DNA methylation levels [12]. These results indicate that only a small fraction (3.9%) of the sites are influenced by lymphocyte counts, and no sites are correlated with the counts of the other cell types. Since we are using samples from a healthy population based cohort in combination with the fact that age-dependent differential methylation is shared between cell types cells [31], the variability in WBC sub-types is not likely to exert a major effect on our results on the effect of chronological age on DNA methylation.

Many age-related diseases, such as coronary artery disease, atherosclerosis, and chronic inflammation are tightly linked to inflammatory processes, were white blood cells play an important role. Variability in hundreds of genes contributes to the susceptibility of these diseases. Altered DNA methylation pattern in blood cells might result in variation in gene transcription and subsequent increased or decrease of the amount of gene product. This regulation might play an important role in the inflammatory process, when the immune cells release a large number of inflammatory markers. It is reasonable to believe that signal molecules that are released as an indication of inflammation might be up- or down- regulated due to variation in the DNA methylation pattern, resulting in an accelerated (or decelerated) immune response to a pathogen, or even a response without being triggered by a pathogen. A large fraction of hypermethylated CpG sites in elderly might result in repression of genes that are important for maintaining health. A better understanding of the changes in DNA methylation through a person’s lifespan might lead to better prevention and treatment of age-related diseases. The strong effect of age on DNA methylation could also introduce a bias in studies of the DNA methylation pattern in diseases, when cases and controls are not carefully age-matched, a factor that needs to be considered in early study design.

## Supporting Information

Figure S1 **The most enriched molecular functions among hypermethylated CGIs.** The colors in the figure represent the level of significance with white = >10^−3^, yellow = 10^−3^ to 10^−5^, orange = 10^−5^ to 10^−7^, dark orange 10^−7^ to 10^−9^, red = <10^−9^.
(PNG)

Figure S2 **The most enriched biological processes among hypermethylated CGIs.** The colors in the figure represent the level of significance with white = >10^−3^, yellow = 10^−3^ to 10^−5^, orange = 10^−5^ to 10^−7^, dark orange 10^−7^ to 10^−9^, red = <10^−9^.
(PNG)

Figure S3 **The most enriched molecular functions among hypomethylated CGIs.** The colors in the figure represent the level of significance with white = >10^−3^, yellow = 10^−3^ to 10^−5^, orange = 10^−5^ to 10^−7^, dark orange 10^−7^ to 10^−9^, red = <10^−9^.
(PNG)

Figure S4 **The most enriched biological processes among hypomethylated CGIs.** The colors in the figure represent the level of significance with white = >10^−3^, yellow = 10^−3^ to 10^−5^, orange = 10^−5^ to 10^−7^, dark orange 10^−7^ to 10^−9^, red = <10^−9^.
(PNG)

Table S1 **Association results for chronological age and level of methylation for all DNA methylation sites.**
(ZIP)

Table S2 **The most enriched molecular functions (FDR P-value<0.0001) among hypermethylated CGIs.**
(XLSX)

Table S3 **The most enriched biological processes (FDR P-value<0.0001) among hypermethylated CGIs.**
(XLSX)

Table S4 **The most enriched molecular functions (FDR P-value<0.0001) among hypomethylated CGIs.**
(XLSX)

Table S5 **The most enriched biological processes (FDR P-value<0.0001) among hypomethylated CGIs.**
(XLSX)
