# Supplementary figures and images for: Continuous Aging of the Human DNA Methylome Throughout the Human Lifespan
Source: PLoS One. 2013 Jun 27;8(6):e67378. doi: 10.1371/journal.pone.0067378 (PMC3695075; doi:10.1371/journal.pone.0067378)

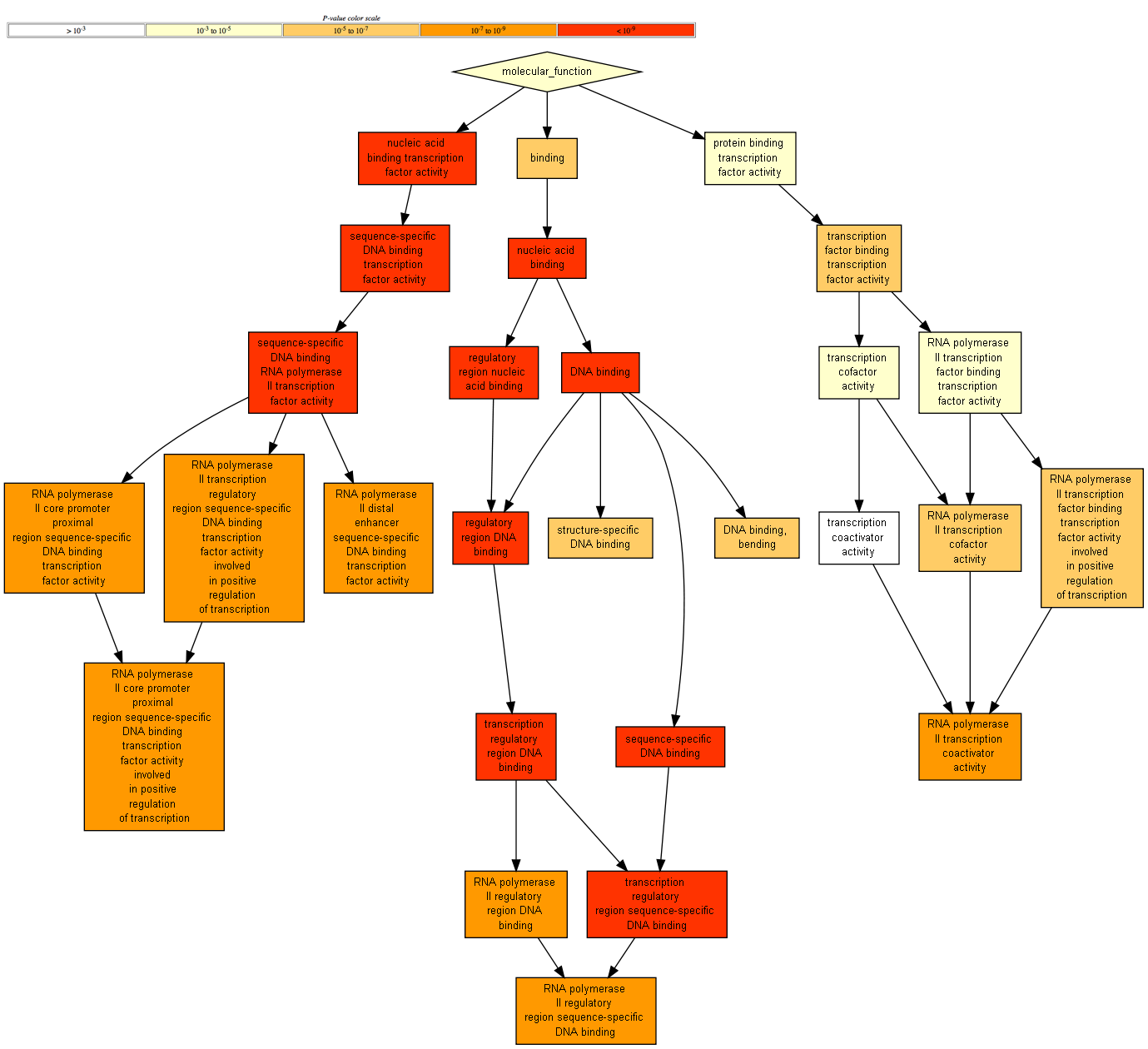

Supplement: Figure S1 — The most enriched molecular functions among hypermethylated CGIs. The colors in the figure represent the level of significance with white = >10−3, yellow = 10−3 to 10−5, orange = 10−5 to 10−7, dark orange 10−7 to 10−9, red = <10−9. (PNG) [file pone.0067378.s001.png]

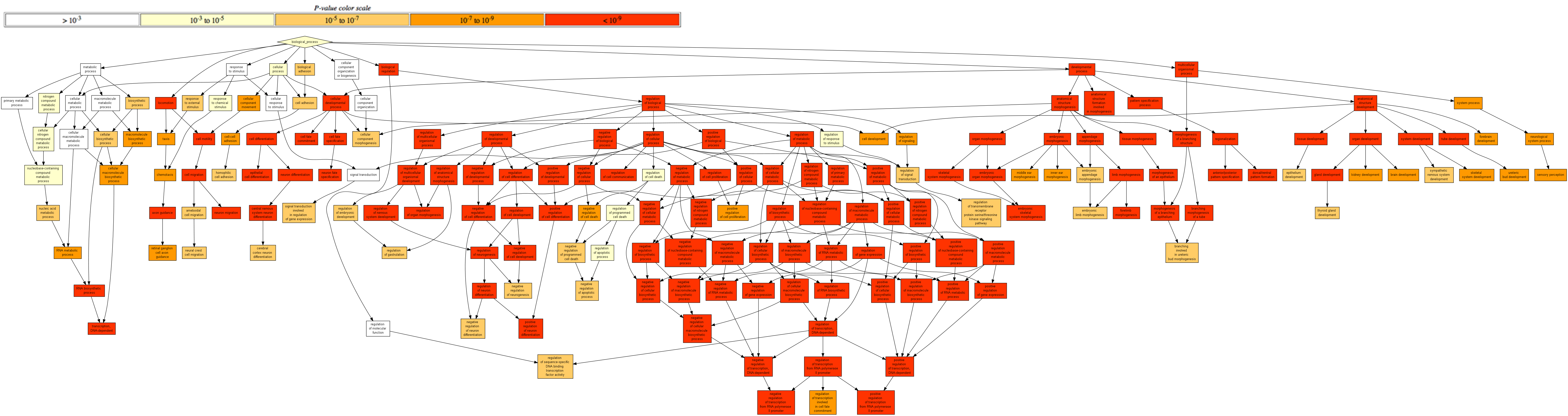

Supplement: Figure S2 — The most enriched biological processes among hypermethylated CGIs. The colors in the figure represent the level of significance with white = >10−3, yellow = 10−3 to 10−5, orange = 10−5 to 10−7, dark orange 10−7 to 10−9, red = <10−9. (PNG) [file pone.0067378.s002.png]

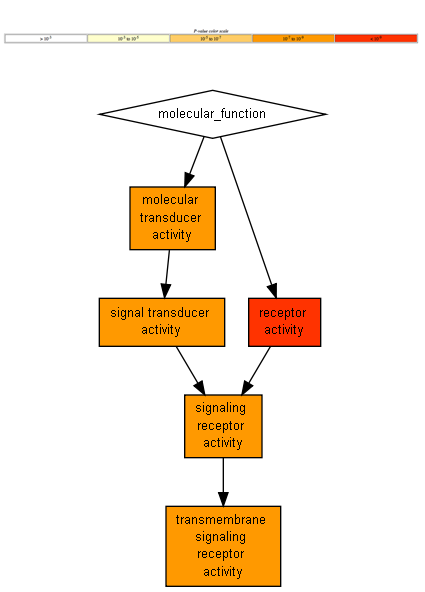

Supplement: Figure S3 — The most enriched molecular functions among hypomethylated CGIs. The colors in the figure represent the level of significance with white = >10−3, yellow = 10−3 to 10−5, orange = 10−5 to 10−7, dark orange 10−7 to 10−9, red = <10−9. (PNG) [file pone.0067378.s003.png]

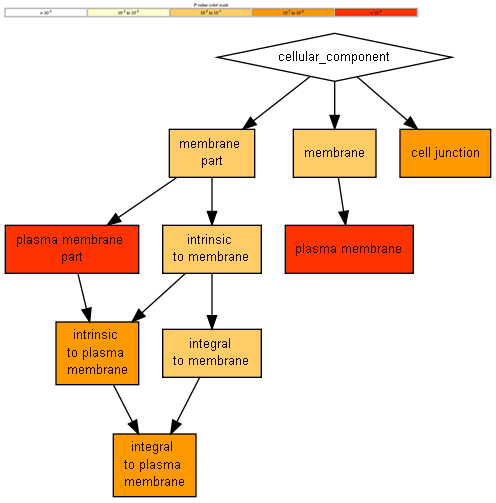

Supplement: Figure S4 — The most enriched biological processes among hypomethylated CGIs. The colors in the figure represent the level of significance with white = >10−3, yellow = 10−3 to 10−5, orange = 10−5 to 10−7, dark orange 10−7 to 10−9, red = <10−9. (PNG) [file pone.0067378.s004.png]
